# A Phase Ib Study of Chemoimmunotherapy with Pegylated Liposomal Doxorubicin and Pembrolizumab in Estrogen Receptor–Positive Metastatic Breast Cancer

**DOI:** 10.1158/2767-9764.CRC-25-0539

**Published:** 2026-07-21

**Authors:** Alberto A. Gabizon, Hadar Goldvaser, Adar Yaacov, Ora S. Rosengarten, Nathan Cherny, Rut Isacson, Shani Breuer, Areen Abu-Remilah, Hilary Shmeeda, Eliahu Golomb, Yehonatan N. Turner, Albert Grinshpun

**Affiliations:** 1 Shaare Zedek Medical Center, Hebrew University-Faculty of Medicine, Jerusalem, Israel.; 2 Memorial Sloan Kettering Cancer Center, New York, New York.

## Abstract

**Purpose:**

Pegylated liposomal doxorubicin (PLD) is a potent immunogenic cell death inducer, allowing for improved tumor-immune recognition and T-cell activation. We report a phase Ib study of combined PLD and pembrolizumab (PEM) in estrogen receptor (ER)-positive/human epidermal growth factor receptor 2–negative metastatic breast cancer (MBC).

**Patients and Methods:**

Patients with MBC, who progressed on hormonal, biological, and cytotoxic chemotherapy, were eligible. Study objectives were safety, response, survival, and pharmacokinetics (PK). The study consisted of two cohorts: PLD 30 mg/m^2^ once every 3 weeks and PLD 40 mg/m^2^ once every 4 weeks, with PEM 200 mg once every 3 weeks in both cohorts. Responding and stable patients continued treatment until disease progression or intolerance.

**Results:**

Thirty-five patients were enrolled and received a total of 201 PLD and 257 PEM treatments. Treatment was well tolerated with no significant neutropenia, no cardiac events, and minimal hair loss. Treatment-related serious adverse events were observed in three patients. In patients receiving >3 cycles, cutaneous toxicity often forced treatment delays. The disease control rate was 67%, including 10 responses with median duration of 11 months. Responses of large liver metastases were observed. The median overall survival was 25 months. PLD PK was monoexponential with high peak plasma concentration, long half-life (∼3 days), slow clearance, and small volume of distribution in the central compartment. PEM plasma levels indicated mean half-life of ∼11 days with high trough concentrations. Gene expression tumor profiling identified 16 genes upregulated in responders versus nonresponders, including interferon-stimulated genes.

**Conclusions:**

The combination of PLD with PEM is well-tolerated, active, and feasible for extended treatment with durable responses. The results suggest possible contribution of PEM to the antitumor effect.

**Significance:**

The combination of PLD and PEM in ER-positive MBC resulted in durable antitumor responses and median survival exceeding 2 years in a heavily pretreated patient group. These clinical observations together with the pharmacologic rationale and gene expression data have translational relevance supporting further exploration of combinations of nanomedicines with immunotherapy in this breast cancer population.

## Introduction

Immune checkpoint inhibitors, such as anti-PD1 antibodies, represent a tremendous quantum leap of progress in cancer therapy. However, except for a few instances of highly immunogenic tumors, combination of immunotherapy with other modalities, particularly cytotoxic or biological therapies, is required for a major improvement of outcomes in most malignancies. In the case of breast cancer, chemoimmunotherapy is effective in triple-negative breast cancer (TNBC). However, in hormone receptor–positive cancers, particularly in the metastatic stage, it is generally accepted that the addition of immunotherapy does not have a significant impact.

In the neoadjuvant setting, addition of pembrolizumab (PEM), an anti-PD1 antibody, to chemotherapy followed by adjuvant PEM improved outcomes in patients with high-risk, early-stage TNBC (1). In the inoperable or metastatic setting, a large phase III study (Keynote-355) investigating first-line therapy in patients with TNBC with a combined positive score (CPS) ≥10 demonstrated significantly longer median progression-free survival (PFS) and overall survival (OS) with PEM and chemotherapy than with placebo and chemotherapy [HR = 0.65 (95% CI, 0.49–0.86) and HR = 0.73 (95% CI, 0.55–0.95), respectively; refs. 2, 3]. In another large phase III study in patients with metastatic TNBC on second- or third-line therapy (Keynote-119), single-agent PEM did not confer a significant prolongation of OS over chemotherapy only, even in patients with CPS ≥10 [HR = 0.78 (95% CI = 0.57–1.06); ref. 4]. Anti-PDL1 antibodies have also been tested in two phase III studies comparing atezolizumab and paclitaxel with placebo and paclitaxel in first-line therapy of patients with metastatic TNBC (IMpassion 130 and 131). In both studies, there was no statistical improvement of OS. In the IMpassion 130 study, an exploratory subgroup of PDL1-positive patients seemed to have an improved outcome with atezolizumab, but this could not be confirmed in IMpassion 131 (5, 6). These studies inform us that early use of immunotherapy and the antibody class (anti-PD1 or anti-PDL1) may be important and that combining chemotherapy is required for best outcomes. However, one interesting point is that the chemotherapy component did not include doxorubicin or other anthracyclines in these studies (3), although doxorubicin is known to be a potent immunogenic cell death (ICD) inducer whereas paclitaxel is not (7, 8).

Two recent studies in the neoadjuvant setting on high-risk, early-stage, estrogen receptor–positive (ER+), and human epidermal growth factor receptor 2–negative (HER2−) breast cancer have reported a significantly greater rate of pathologic complete response (CR) when PEM or nivolumab was added to a chemotherapy protocol consisting of paclitaxel, doxorubicin/epirubicin, and cyclophosphamide (9, 10). Longer observation time is required to assess further endpoints.

In patients with ER+, HER2−, metastatic breast cancer (MBC), the addition of PEM to eribulin did not improve PFS, objective response rate, or OS compared with eribulin alone in a randomized phase II clinical trial (11). In another randomized phase II study in ER+, HER2− MBC, the effect of low-dose pegylated liposomal doxorubicin (PLD) and oral cyclophosphamide, referred to as “immunomodulatory” chemotherapy with or without ipilimumab and nivolumab, was examined: no significant improvement in the primary endpoint, median PFS, was observed (12).

There is scarce and only early-phase clinical data on the use of a nanomedicine, PLD (PLD is known also by the innovator product names, Doxil or Caelyx), as the chemotherapy arm of chemoimmunotherapy protocols of cancer therapy (13–15). Specifically, in the context of MBC, there is only the above-mentioned study (12).

**Figure 1.**
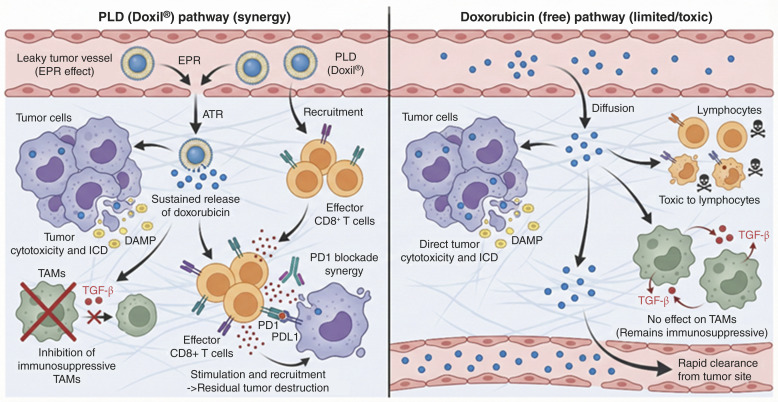
Rationale for PLD (Doxil) in chemoimmunotherapy—a comparative diagram of the interactions of PLD and free doxorubicin taking place in the tumor site. Following tumor delivery by way of EPR and accelerated transport and release (ATR), PLD exerts indirect and direct effects on tumor cells mediated by sustained release, inhibition of immunosuppressive TAMs, tumor cytotoxicity, release of danger signal molecules (DAMP), and ICD, resulting in stimulation and recruitment of effector CD8^+^ T cells which will destroy residual tumor cells in synergy with PD1 blockade. Doxorubicin tissue access by diffusion leads to a direct tumor cytotoxic effect and ICD but is also toxic to lymphocytes, will not affect TAMs, and is rapidly cleared from the tumor site.

PLD contains doxorubicin, a potent ICD inducer (16), has a low myelosuppressive and immunosuppressive profile, as well as a favorable cardiac safety profile compared with doxorubicin. In addition, it has the unique pharmacologic attributes of stealth nanomedicines which include slow drug release and passive tumor targeting (17). We hypothesized that a PLD-based therapy should allow for improved immune recognition of tumor cells and activation of T cells by anti-PD1 blockade (Fig. 1; ref. 18). The choice of patients with ER+, HER2− tumors was aimed at addressing the major subgroup of patients with breast cancer in whom immunotherapy is considered less effective. Additional factors supporting the use of PLD rather than free doxorubicin are the demonstrated noninferiority of PLD in MBC and its much-improved safety profile, with a major cardiac sparing effect (19, 20). Furthermore, PLD has been tested in anthracycline-naïve and pretreated patients with apparently similar efficacy as measured by the clinical benefit rate in patients with a favorable performance status (21). Finally, in animal studies, PLD reduced the tumor-associated macrophage (TAM) population by 50% as compared with free doxorubicin (22); in addition, in two mouse tumor models, PLD demonstrated both anticancer efficacy and synergy with immune checkpoint blockade in immunocompetent mice, whereas doxorubicin did not (23, 24). Collectively, these observations (ICD effect, passive tumor targeting, sustained *in situ* drug release, and suppression of tumor growth–promoting TAM) suggest that PLD has a unique value in chemoimmunotherapy (17).

Based on this rationale, we undertook this single-center phase Ib study to examine the safety, efficacy, and pharmacokinetics (PK) of a combined regimen of PEM, an anti-PD1 antibody, and PLD in the treatment of patients with endocrine–resistant, ER+ MBC.

## Patients and Methods

### Study design

Female patients, ages 18 years and above, with Eastern Cooperative Oncology Group (ECOG) performance score between 0 and 2; pathologic diagnosis of ER+, HER2− breast cancer; stage IV disease; whose disease progressed on hormonal, CDK 4/6 inhibitors; and up to three lines of chemotherapy were eligible for enrollment to the study.

The study consisted of two cohorts: a first cohort of 15 patients in which PLD was infused at a dose of 30 mg/m^2^ and PEM at a flat dose of 200 mg every 3 weeks, and a second cohort of 20 patients in which PLD was infused at a dose of 40 mg/m^2^ every 4 weeks and PEM at a flat dose of 200 mg every 3 weeks. Cohort 2 was added under an amendment of the study to bring the dose of PLD to a level considered more appropriate when PLD is given without additional cytotoxic agents and pending that no dose-limiting toxicities were observed in the first cohort. See the study diagram in Supplementary Fig. S1.

Adverse events (AE) and serious AEs (SAE) were registered and graded in accordance with the National Cancer Institute Common Terminology Criteria for AEs (CTCAE version 4.03). On predefined time points around each dosing, safety laboratory assessments were done [blood chemistry, urine analysis, and hematology; the full schedule of assessments can be found in the Supplementary File Protocol (clinical study protocol: KEYDOX, MK3475-733)].

The primary study objectives were to establish the safety of the PLD–PEM combination regimen in all patients treated and to evaluate the tumor response rate in patients undergoing reevaluation after the three first cycles of treatment. Secondary objectives were to characterize the PK profile of PLD and PEM when delivered in combination and to measure the duration of response, PFS, and OS.

For responding or stable patients, the extended phase of the study consisted of nine additional cycles during which safety information, duration of response, PFS, and survival data were collected. Further on, treatment continuation was at physician’s discretion. Further data were collected on safety events and PFS for patients continuing study therapy and on post-study treatments and survival for all patients until data lock on December 2025.

The study (acronym: KEYDOX) was conducted in accordance with the ethical guidelines of the Declaration of Helsinki under approval by the Ethics Committee of Shaare Zedek Medical Center (310-17-SZMC) and was opened on April 2019. Written informed consent was obtained from all patients. For the study protocol, go to ClinicalTrials.Gov, study identifier NCT03591276.

### Treatment administration

PLD (Caelyx or generics) was provided by the Hospital Pharmacy. PEM (Keytruda) was provided by MSD Israel. Both drugs were infused on study day 1 in our day care ambulatory center. Prior to PLD infusion, patients received premedication consisting of indomethacin 25 to 50 mg or ibuprofen 400 mg, fexofenadine 180 mg, and famotidine 20 mg orally at least 1 hour before and 4 to 8 mg dexamethasone intravenously immediately prior to PLD infusion. Metoclopramide 10 mg intravenously or palonosetron 0.25 mg intravenously were optional at the physician’s discretion. The target maximal rate of PLD infusion was up to 1 mg/minute, and the start rate was not greater than 0.25 mg/minute with gradual stepwise increases every 15 minutes. If the first couple of treatments went through uneventfully, premedication was de-escalated and the target rate of infusion was reached with fewer incremental steps, thus shortening the infusion time. PEM was infused in 30 minutes as directed.

### Safety evaluation

Patients were examined weekly during the first three cycles and evaluated by the primary care physician for side effects or clinical deterioration at the beginning of every cycle. AEs and SAEs were graded by the NCI CTCAE, as indicated previously.

### Efficacy evaluation

After every three cycles, patients underwent disease reevaluation by CT or PET-CT. Responses were evaluated by Response Evaluation Criteria in Solid Tumors, version 1.1, whenever possible, and by PET-CT evaluation of the lesion extension along with the fluorodeoxyglucose (FDG) uptake area and the maximum Standardized Uptake Value (SUVmax). Blood tumor markers (carcinoembryonic antigen, CA15-3, CA125) were measured once per cycle during the first three cycles, and optionally thereafter.

### PK analysis of plasma levels of PLD and PEM

Blood samples (∼3 mL) were obtained from 24 patients in the first and third cycles and were withdrawn into vacuum-sealed K-EDTA–containing tubes before infusion and at the following time points after the end of infusion: 1 hour, 3 hours, 24 hours, 3 to 4 days, 7 ± 1 day, 14 ± 1 day, and 21 ± 1 day. Plasma was separated from blood cells by centrifugation and stored at −80°C. The doxorubicin plasma levels following infusion of PLD were analyzed by high-performance liquid chromatography with fluorometric detection at ex: 470/em: 590 nm wavelength, as previously described (25). Daunorubicin served as the internal standard. The concentration of doxorubicin was calculated based on the relative peak areas of doxorubicin and the daunorubicin internal standard. This method can detect doxorubicin within a range of 10 ng to 5,000 ng/mL. The drug measured represents the total amount of drug in plasma, including the liposomal fraction, protein-bound fraction, and free fraction. However, previous studies of PLD PK have shown that >95% of the doxorubicin measured in plasma is liposome-bound, and any released doxorubicin is cleared at a rate several hundred-fold faster than liposomal drug. Therefore, the total doxorubicin plasma level can be considered representative of the PK of total PLD and of the liposomal drug fraction (26, 27).

PEM plasma concentrations were analyzed using the Pembrolizumab ELISA Kit ab237652 (Abcam, RRID: SCR_012931) following the instructions therein.

The following PK parameters were calculated for plasma concentrations of doxorubicin and PEM by nonlinear least squares curve fitting using GraphPad Prism software version 10 (RRID: SCR_002798).

- C_max_ (mg/L), peak plasma concentration.
- T½ (hours), terminal half-life.
- AUC (mg × hour/L), area under the plasma concentration versus time curve calculated using the trapezoidal method from time zero to the actual time corresponding to the last concentration above the limit of quantification (AUC_0–last/21 days_).
- CL (L/hour), systemic clearance calculated as CL = dose/AUC.
- V_cc_ (L), apparent volume of distribution in the central compartment calculated as dose/C_max_.
- C_trough_ (mg/L), the trough concentration at the end of the cycle (day 21).

### Statistical methods

Data were tabulated in Excel files, and all statistical analyses were done using GraphPad Prism version 10 (RRID: SCR_002798). Statistical significance of differences between means was analyzed by the parametric unpaired or paired *t* tests and, in a few instances, by the nonparametric Mann–Whitney or Wilcoxon signed-rank tests. All *P* values are two-tailed. Correlations were analyzed by linear regression and Pearson or Spearman correlation coefficients. The Kaplan–Meier test was used for analysis of PFS and OS curves. The median follow-up time was estimated by reverse Kaplan–Meier analysis. Survival times were measured in days and converted in months using a month average value of 30 days.

### Biomarkers

In a subgroup of patients, information was available on mismatch repair (MMR) or microsatellite instability status examined by immunohistochemistry (IHC) or PCR respectively; PDL1 expression examined by IHC and quantified by the combined pathologic score (CPS); and tumor mutation burden (TMB) analyzed by next-generation sequencing.

In addition, in a group of 12 patients with adequate tumor tissue samples, including six patients identified as responders and six patients as nonresponders to treatment, we conducted gene expression profiling using the immuno-oncology (IO) 360 panel (750 genes) of the NanoString: nCounter Analysis System (RRID: SCR_021712). Data were normalized using positive control and housekeeping gene normalization, followed by log_2_ transformation. Differential expression was assessed using theWelch *t* test with significance thresholds of unadjusted *P* < 0.05 and |log_2_ fold change| > 0.5.

## Results

### Patient characteristics

Thirty-eight patients signed informed consent, but two patients failed screening and one withdrew consent, leaving 35 treated patients evaluable for survival and safety. Nearly all patients (32/35) had visceral disease. Five patients did not reach the third cycle due to clinical deterioration (one of these five patients was withdrawn because of active gastrointestinal bleeding diagnosed during the course of the first infusion of PLD. She did not receive PEM but recovered quickly and responded to PLD, which was then continued off-study), leaving 30 patients evaluable for antitumor response. Post-study therapy was mainly based on cytotoxic agents. In three cases, patients received antibody–drug conjugates (ADC). None of the patients were known to be carriers of the three common germline mutations of *BRCA1/2* genes. Table 1 presents the characteristics of the patient population and treatment received.

**Table 1.** Characteristics of the study population.

Characteristic						
*N* patients^a^	35					
Age (years):	​					
Mean (95% CI)	61.2 (57–65.4)					
Median (range)	60 (36–91)					
Weight (kg):	​					
Mean (95% CI)	69.9 (63.7–76)					
Median (range)	65.3 (44–120.6)					
BMI (kg/m^2^):	​					
Mean (95% CI)	26.9 (25–28.9)					
Median (range)	25.9 (18.6–40.1)					
*N* IDC/ILC/IDC + ILC	30/4/1					
*N* ER+/HER2−^b^	35 (100%)					
ECOG PS	0		1		2	
* N* patients	3​		17​		15​	
*N* chemotherapy lines^c^	0	1	2	3	4	Doxorubicin yes/no
* N* patients	1	7	11	12	4	22/13
Total *N* courses given	PLD: 203			PEM: 259		
* N* per patient, median (range)	PLD: 3 (1–21)			PEM: 5 (0–30)		
Post-study treatment	Palliative only			Cytotoxics	Hormonal–biological	ADC
* N* patients	8			26	1	3

Abbreviations: ACT, doxorubicin-cyclophosphamide-paclitaxel regimen; BMI, body mass index; LHRH, lutein hormone-release hormone; PS, performance score.

a All patients received prior hormonal therapy (estrogen receptor modulators, estrogen receptor degraders, aromatase inhibitors, and/or LHRH analogs) and biological therapy (CDK4/6 inhibitors, everolimus, and/or PI3K inhibitors).

b HER2 by IHC: 0, 1, or 2, the latter confirmed negative by FISH.

c Chemotherapy lines include neoadjuvant and adjuvant chemotherapy. Neoadjuvant or adjuvant ACT was considered 1 line. In four patients, a waiver was given to enter the study despite having received four chemotherapy lines (instead of a maximum of three as specified in the protocol).

### Safety

Overall, treatment was well tolerated. Low-grade (grades 1–2) fatigue and lack of appetite were the most common AEs. Nausea and vomiting, myelosuppression, and mucositis were infrequent and limited to grades 1 to 2. There was no significant hair loss (grades 0–1). Skin toxicity in the form of palmar–plantar erythema (PPE) was observed after two or more cycles not exceeding grade 2 but forcing treatment delays of 1 to 2 weeks in three patients of the first cohort. It was one of the reasons for switching to a 4-week dose interval in the second cohort. Other forms of skin toxicity observed were a pressure sore in the ankle, grade 2, probably PLD-related, forcing delay of treatment in one patient, and a rash in extremities, grade 1 to 2, possibly PEM-related. There was no clinical evidence of cardiac toxicity. There were two instances of acute infusion reaction to PLD in the same patient, grades 2 and 3, not life-threatening but forcing discontinuation of PLD in the second cycle.

There were five cases of low grade, mostly subclinical, hypothyroidism, probably related to autoimmune thyroiditis as a side effect of PEM, which resolved quickly to reference laboratory values with levothyroxine oral treatment. We recorded 10 SAEs in nine patients, seven of which were disease-related and not treatment-related, including an early death in the first 30 days in study probably related to progressive disease (PD) and hepatorenal failure. There were three SAEs possibly or probably related to treatment: two cases of hepatitis with steep increases of transaminases and lactate dehydrogenase (grade 4) and upper abdominal symptoms and one case of grade 4 hemolytic anemia. All three patients discontinued PEM, as well as PLD, and recovered with corticosteroid treatment in the hepatitis cases and corticosteroids with rituximab treatment in the case of hemolytic anemia. A descriptive list of SAEs is presented in Supplementary Table S1.

### Efficacy

**Figure 2.**
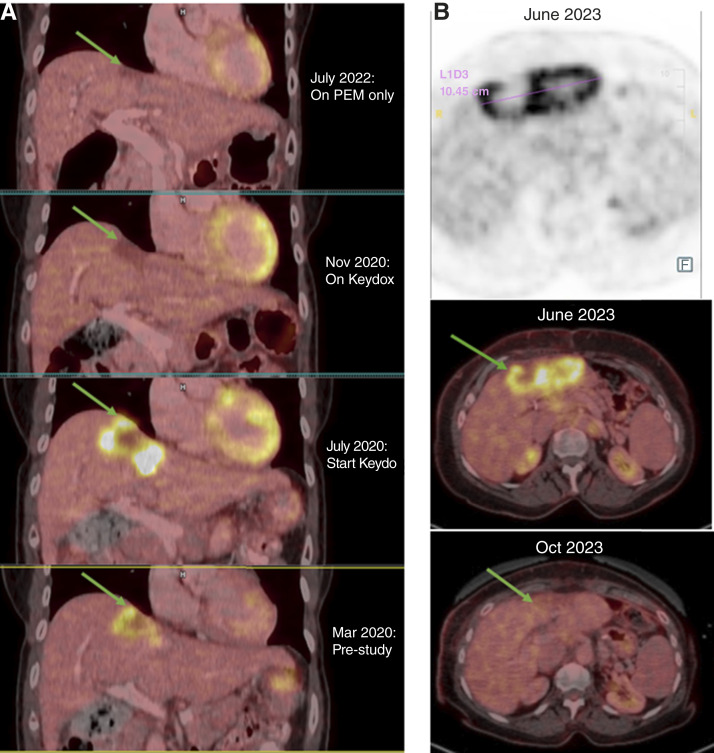
Response of hepatic metastasis to PLD + PEM in patients #10 (**A**) and #30 (**B**). **A,** This 75-year-old patient (see #10 in Supplementary Table S3; Supplementary Fig. S3) with a large liver metastasis had a complete and durable response. Bottom to top, PET-CT fusion images of a metastasis with significant progression between March and July 2020 (green arrows), followed by a major response in November 2020 under the Keydox regimen and a residual scar on July 2022 under single-agent PEM for the last 9 months. **B,** This 65-year-old patient (see #30 in Supplementary Table S3; Supplementary Fig. S9) with a large liver metastasis had a complete and durable response. Top to bottom, PET captures and fusion images of a 10.45-cm metastasis (pink line) at start of Keydox treatment (green arrow) with a complete regression 4 months later, maintained with PEM-only treatment for 12 months.

Thirty patients reached the third cycle and were reimaged with CT or PET-CT to evaluate antitumor response. Upon disease reevaluation, the disease control rate (DCR) was 67% (20/30 patients). Ten patients (33%) had functional/metabolic (based on FDG uptake) and/or morphologic (CT scan) responses (Table 2). Interestingly, many of these responses were observed in patients with advanced metastatic liver disease and were unusually striking by PET imaging, indicating rapid and near-complete metabolic shutdown of tumors which reached up to 10.5 cm longest diameter (see Fig. 2; Supplementary Figs. S2–S11). In two of these patients (Fig. 2A and B), the responses were prolonged CRs interrupted by the appearance of new bony metastasis or new lymphadenopathy without liver relapse. Furthermore, in both patients the response was maintained with only PEM treatment for 9 and 12 months, without PLD, which was discontinued because of fatigue at the patient’s request and because of an acute infusion reaction to PLD on the second cycle, respectively. The median duration of response was 11 months, with the longest response reaching 20 months. The median OS of responders (39 months) was nonsignificantly longer than that of patients with stable disease (SD; 27 months) and more than four-fold longer than that of patients with PD as best response (9 months; *P* = 0.0010, Gehan test), as seen in Table 2. The prior-to-study and post-study therapies of responders are listed in Supplementary Table S2. In 50% of the responders, there was previous exposure to free doxorubicin, although it was in the frame of neoadjuvant/adjuvant treatment in all cases. None of the responders received ADCs as post-study therapy.

**Table 2.** Response rates, duration of response^a^, and survival^b^.

Best response	PR/CR	SD	PD
*N* (%)	10 (33.3%)	10 (33.3%)	10 (33.3%)
Duration (months) (Median, 95% CI)	11 (5–15)	5.5 (3–9)	≤2
Survival (months) (Median, 95% CI)	39 (12–undetermined)	27.3 (8–38.2)	9.2 (2.5–12)
Survival of PR/CR vs PD (Gehan test)	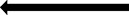 *P* = 0.0010 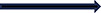		

a Measured from the time of evaluation at cycle 3 till first the evidence of PD.

b Measured from day 1 of cycle 1 until death from any cause. Median estimated by Kaplan-Meier analysis.

**Figure 3.**
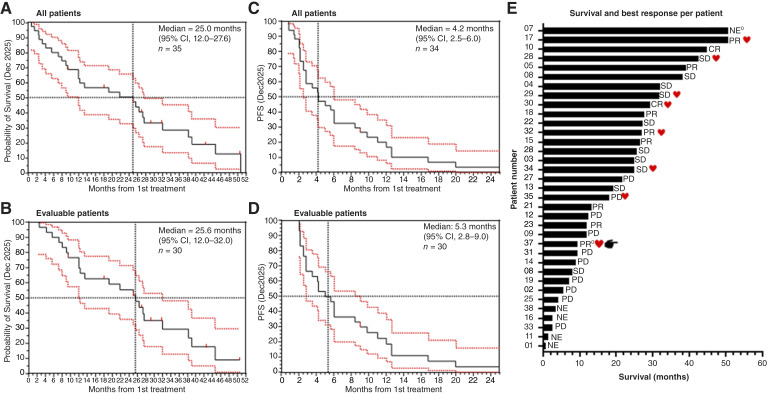
**A–E,** Survival analysis. **A–D,** Kaplan–Meier survival and PFS curves of all patients (**A** and **C**) and evaluable patients (**B** and **D**). **E,** Survival waterfall plot of all patients with best response labels: CR, PR, SD, PD, and NE (patients who did not reach the third cycle of PEM). A heart symbol denotes patient still alive at data lock. (a) In patient 7, the PEM infusion was aborted because of acute GI bleeding. She was considered NE and went on to receive PLD only. (b) Patient 37 was still treated within the Keydox study at data lock.

Survival and PFS curves by Kaplan–Meier analysis of the whole patient population (*n* = 35) and of the evaluable group (*n* = 30) are presented in Fig. 3A–D respectively. The median follow-up was 45.6 months. The median OS for all patients entering the study is 25 months, and the median PFS is 4.2 months. For the evaluable patients (*n* = 30), the median OS is 25.6 months and median PFS is 5.3 months. Figure 3E depicts individual patient survival with best response in a waterfall plot form. There was a nonstatistically significant numerical advantage in OS for the second cohort (PLD = 40 mg/m^2^) when compared with the first cohort (PLD = 30 mg/m^2^). The OS of the first cohort was 15.8 months (95% CI, 5.6–32) and the OS of the second cohort was 25.6 months (95% CI, 7.2-not reached). However, given that the two cohorts were not run in parallel, but sequentially, post-study therapies may also have contributed to this trend, especially as the median PFS of both cohorts were very similar [first cohort = 3.9 months (95% CI, 2–9); second cohort = 4.2 months (95% CI, 2.5–9.8)]. Eight patients (three previous responders, one responder still on treatment, three patients with previous SD, and one patient with PD) remain alive at data lock with a survival time in the range of 9.6+ to 50.6+ months.

As expected, the ECOG score was highly prognostic of survival, with higher score faring worse than lower score (Spearman’s coefficient: −0.62; *P* < 0.0001). Age and body mass index were not correlated with survival. In the responder group, the duration of response was positively correlated with survival (Spearman *r* = 0.81, *P* = 0.0064; Pearson *r* = 0.77, *P* = 0.0089; Supplementary Fig. S12). Prior exposure to free doxorubicin did not have any deleterious effect on survival (Fisher exact test risk ratio: 1.025; 95% CI, 0.62–1.94, for 1-year risk of death,), nor did it seem to interfere with the chance of response, as suggested by an equal number of responders with or without prior doxorubicin (Supplementary Table S2). However, as most patients who were pretreated with doxorubicin received the drug in a neoadjuvant or adjuvant setting, it is possible that residual drug resistance has waned at the time of PLD treatment.

### PK analysis

In agreement with previously published data (28), PLD clearance was best described in most cases by a single exponential and characterized by a high C_max_ (mean >20 mg/L), long T½ (∼2.5 to 3 days), slow clearance (∼25 mL/hour), and small volume of distribution (V_cc_) roughly equivalent to the plasma volume (Table 3). In addition, there was a mean increase in half-life and AUC (∼20%) of PLD when comparing the first with the third cycles (Table 3) confirming previous results (25) and indicating delayed clearance after repeated treatment with PLD. The rise of PLD dose from 30 to 40 mg/m^2^ resulted in a significant increase of the C_max_ and AUC, proportionally similar to the increase of dose (Table 3). The C_max_-average per day, considered a predictor of response to PLD in patients with Kaposi sarcoma were nearly equal for the 30 and 40 mg/m^2^ groups (C_max_-average/day = 1.1 mg/L/day at both dose levels). There was a slightly higher dose intensity in the 40 mg/m^2^ group (dose intensity = 16.9 and 17.5 mg/week for 30 and 40 mg/m^2^ dose levels, respectively).

**Table 3.** PK parameters of PLD [mean (SEM)].

Patient group	Dose mg/m^2^	Cycle (C) #	Given dose mg^a^	C_max_ mg/L^b^	T½ hours^c^	AUC_0–__last_ mg × hour/L^d^	CL mL/hour^e^	V_cc_ L^f^
Cohort 1 (*n* = 14)	30	1	50.7 (1.2)	22.5 (1.7)	71.7 (3.2)	2,472 (204)	22.7 (1.8)	2.4 (0.2)
Cohort 1 (*n* = 12)	30	3	49.5 (1.4)	24.75 (1.5)	81.4 (5.3)	3,086 (269)	17.5 (1.8)	2.1 (0.1)
Cohort 2 (*n* = 10)	40	1	70 (2)	29.5 (3.2)	57.9 (4.1)	3,045 (434)	27.6 (4)	2.5 (0.2)
Cohort 2^g^ (*n* = 3)	40	3	69 (4.6)	24.75 (2.5)	79.3 (8.7)	3,601 (63)	18 (1.5)	2.3 (0.5)

a Cohort 1 - C1 vs. C3, not significant (paired *t*); cohort 1 C1 vs. cohort 2 C1, *P* < 0.0001 (unpaired *t*).

b Cohort 1 - C1 vs. C3, *P* = 0.0068 (paired *t*); cohort 1 C1 vs. cohort 2 C1, *P* = 0.0162 (unpaired *t*).

c Cohort 1 - C1 vs. C3, borderline significant *P* = 0.0698 (paired *t*); cohort 1 C1 vs. cohort 2 C1, *P* = 0.0131 (unpaired *t*).

d Cohort 1 - C1 vs. C3, *P* = 0.0008 (paired *t*); cohort 1 C1 vs. cohort 2 C1, not significant (unpaired *t*).

e Cohort 1 - C1 vs. C3, *P* = 0.0015 (paired *t*); cohort 1 C1 vs. cohort 2 C1, not significant (unpaired *t*).

f Cohort 1 - C1 vs. C3, *P* = 0.0184 (paired *t*); cohort 1 C1 vs. cohort 2 C1, not significant (unpaired *t*).

g Cohort 2 C3 is a small group (*n* = 3) and was not tested by statistical analysis. Numerically, the results of AUC and CL were consistent with the observations in cohort 1 C3.

Doxorubicinol levels were variable and borderline detectable (≤1% of doxorubicinol level), suggesting that the slow clearance of liposomal drug reduces the rate of metabolite production below its clearance rate as previously observed (26).

**Figure 4.**
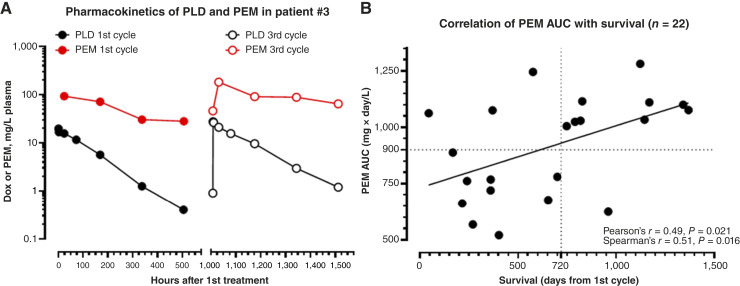
**A,** Plasma clearance of PLD and PEM in first and third treatment cycles of patient #3. Note the high trough level of PEM and the higher AUC of PLD in the third cycle. In both cases, clearance is slow and monoexponential. Dox, doxorubicin. **B,** Correlation of PEM AUC and survival by linear regression analysis. Patients with AUC < 900 had a significantly lower probability reaching a survival of ≥720 days (1/10) than patients with AUC > 900 (9/12). Fisher exact test RR = 0.13 (95% CI, 0.02–0.58), *P* = 0.0037.

The PEM half-life was ∼11 days with nearly ∼100% of the injected dose still in plasma at 24 hours and high trough concentrations by the end of the treatment cycle (Table 4). PEM is mostly retained in the central vascular compartment, as indicated by the small V_cc_ values. The increased dose of PLD in the second cohort did not have any impact on the PK of PEM. In two patients, we followed the plasma levels of PEM after the first and third cycles and found a major increase in C_max_, AUC and C_trough_ (Supplementary Table S3). This is not surprising given that the half-life of PEM is relatively long for a 3-week dose interval to allow for full clearance. It does, however, suggest that most of the accessible PD1 receptors are probably saturated. A comparative plot of the plasma clearance of PLD and PEM (Fig. 4A) illustrates head-to-head the clearance kinetics of both compounds.

**Table 4.** PK parameters of PEM at a flat dose of 200 mg.

Mean (SEM)	C_max_ mg/L	AUC_0–__last_ mg × day/L	T½ days	CL mL/day	V_cc_ L	C_trough_ (day 21) mg/L
All patients (*n* = 22)	88.4 (6.5)	929.4 (45)	11.75 (0.8)	227.7 (12.7)	2.5 (0.2)	26.65 1.7
Cohort 1^a^ (*n* = 12)	96 (9.5)	970.6 (53.1)	11.5 (1.2)	214.3 (13.8)	2.3 (0.2)	28 (2.3)
Cohort 2^b^ (*n* = 10)	79.3 (8.1)	880 (75.85)	12.1 (1.1)	243.7 (22.2)	2.8 (0.3)	25.1 (2.5)

a Received PEM in combination with PLD = 30 mg/m^2^.

b Received PEM in combination with PLD = 40 mg/m^2^.

We next tested whether PK factors are correlated with patient outcome. There were no statistically significant correlations between the C_max_ or AUC of PLD during the first and third cycles, with survival or response. However, we found that the initial dose of PLD was positively correlated with survival when expressed as mg/kg weight of given dose per cycle (Pearson coefficient: +0.49, *P* = 0.03). This is consistent with previous reports indicating that dose intensity of PLD is correlated with efficacy (29). Regarding PEM, we tested whether the C_max_, AUC, and trough concentration measured on day 21 (at the end of the cycle) correlated with response or survival. There was a statistically significant positive correlation between the AUC of PEM and survival (Fig. 4B). Patients with AUC < 900 mg × day/L had a significantly lower probability of survival reaching 720 days (∼2 years) than patients with AUC > 900 mg × day/L (Fisher exact test risk ratio: 0.13; 95% CI, 0.02–0.58; *P* = 0.0037). This effect may simply be due to the faster clearance of PEM in patients with advanced, nonresponding disease, as previously reported (30). It is also possible that a higher AUC may enhance the penetration of PEM into tumor tissue by the enhanced permeability and retention (EPR) effect (31).

### Biomarkers

**Figure 5.**
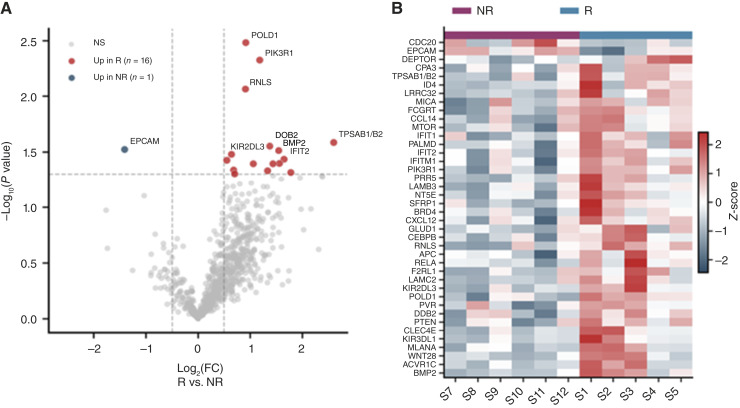
**A,** Volcano plot of differential gene expression between responders (R) and nonresponders (NR). Each point represents a gene. Red points indicate genes significantly upregulated in Rs (*n* = 16; unadjusted *P* < 0.05, log_2_FC > 0.5). Blue point indicates genes upregulated in NRs (*n* = 1; EPCAM). Gray points represent nonsignificant genes. Dashed lines indicate significance thresholds (horizontal: *P* = 0.05; vertical: log_2_FC = ±0.5). Top genes are labeled. **B,** Heatmap of top 40 differentially expressed genes. *Z*-score normalized expression values are shown for NRs (purple, left) and Rs (blue, right). Genes were clustered hierarchically using the Ward method. Red indicates higher expression. Blue indicates lower expression relative to the mean across all samples. For a direct comparison of expression of immune checkpoint genes in Rs vs. NRs, see Supplementary Fig. S13. FC, fold change; NS, not significant.

In a subgroup of 16 patients, we were able to collect information on several biomarkers, including MMR, PDL1, and/or TMB (Supplementary Table S4). The only finding of interest was that one of the patients with CR and longest responders of the study was PDL1-positive (CPS = 17). To examine whether responders and nonresponders to the PLD/PEM combination have a distinct tumor gene expression profile, we conducted a NanoString IO 360 analysis in a subset of 12 patients comprising a group of responders and a group of nonresponders randomly selected and for whom we had adequate tumor tissue samples (Fig. 5). One sample (S6) was excluded because of an extreme housekeeping normalization factor (8.5x), indicating poor RNA quality or low input. Differential expression analysis identified 17 genes altered between responders and nonresponders. Sixteen genes were upregulated in responders, including *POLD1*, *PIK3R1*, *RNLS*, and interferon (IFN)-stimulated genes (*IFIT1*, *IFIT2*, and *IFITM1*). Only EPCAM was upregulated in nonresponders (Fig. 5A). Hierarchical clustering of the top 40 differentially expressed genes showed clear separation between responder and nonresponder groups (Fig. 5B). Immune checkpoint genes (CD274/PDL1, PDCD1/PD1, IDO1, and CD276), particularly CD274/PDL1, showed a trend toward higher expression in responders which did not reach statistical significance (Supplementary Fig. S13).

## Discussion

Immunotherapy has been investigated extensively in TNBC and demonstrated as an effective approach in combination with chemotherapy in neoadjuvant therapy and for first-line therapy of MBC in PDL1-positive patients (32). However, ER+ breast cancer seems to be less susceptible to immunotherapy. This prompted us to explore the use of a combination of PLD and PEM in endocrine–resistant ER+ breast cancer. As already mentioned, PLD was chosen as chemotherapy component because it is mildly myelosuppressive and nonimmunosuppressive and contains doxorubicin, a strong ICD-inducer (17).

The average half-life of PEM observed in this study (11–12 days) is notably shorter than the half-life reported in several studies (20–27 days) for single-agent PEM and for most humanized monoclonal antibodies (33, 34). This may be due to the accelerated catabolic rate of proteins in patients with advanced cancer or inflammatory conditions. In fact, responding patients tend to have a time-variable clearance (slower clearance) of PEM as tumor shrinks and inflammation subsides (30, 33). This effect of tumor burden on clearance may also explain the trend to a shorter survival in patients with smaller AUC of PEM (Fig. 5). Although unlikely, it cannot be ruled out that PLD accelerates the hepatic clearance of PEM, but this needs to be confirmed in a study comparing the PK of PEM with or without PLD co-administration.

All the patients recruited to this study had hormone-refractory MBC and had also failed to CDK 4/6 inhibitors. In addition, they all received one or more chemotherapy lines, which in many cases included prior neoadjuvant or adjuvant treatment with free doxorubicin. The median survival of this heavily pretreated population exceeded 2 years (25 months) and was slightly above that observed with first-line, single-agent PLD in a large study in MBC (21 months; ref. 20). In patients with MBC pretreated with chemotherapy, single-agent PLD has resulted in lower median survival values in the range of 9.6 to 16 months (21, 35–38), far shorter than in the current study, suggesting that the addition of PEM has a positive long-term impact on survival. Survival data of the control chemotherapy group from a recent study with the ADC sacituzumab govitecan in a similar ER+ population points at a median OS of 11.2 months, also far from the 25-month mark observed in this study (39). The combination of PLD and PEM was well tolerated and feasible for prolonged treatment. However, in patients treated with PLD 30 mg/m^2^ every 3 weeks, skin toxicity (PPE) often forced treatment delays from 3 to 4 weeks after three or more cycles of treatment.

The well-accepted regimen of 40 mg/m^2^ once every 4 weeks is in the mid-dose range of linear clearance of PLD (30–60 mg/m^2^; ref. 25) and is more convenient with regard to prevention of PPE than 3-weekly regimens. It also results in a higher C_max_ of PLD than the 30 mg/m^2^ dose level (mean 29.5 vs. 22.5 mg/L, *P* = 0.0162, Table 3), a PK factor correlated in animal studies and patients with Kaposi sarcoma with greater tumor drug delivery and/or efficacy (40). Furthermore, in a small phase II study, the same regimen of PLD 40 mg/m^2^ once every 4 weeks and PEM 200 mg once every 3 weeks in patients with platinum-resistant recurrent ovarian cancer was well tolerated and showed remarkable efficacy (DCR = 52%) in this difficult-to-treat population (14). However, we should still note that there is no evidence of a significant therapeutic advantage over 30 mg/m^2^ once every 3 weeks in the clinical literature.

About 1 of 3 of the patients experienced substantial and often durable PET-CT responses, particularly in patients with liver involvement. These responses are seldom observed with chemotherapy only in a heavily pretreated patient population, as was the case in this study. This is consistent with the hypothesis-generating proposition of a synergistic antitumor effect of the combination of PLD and PEM on liver metastases. The tumor EPR effect (31, 41) together with the intrinsic hepatotropism of nanoparticles and macromolecules such as monoclonal antibodies (42, 43) may both contribute to the antitumor effect of PLD and PEM on liver metastases. Although the microvasculature of tumors and the surrounding sinusoids of normal liver tissue are broadly different anatomically and physiologically, it cannot be ruled out that the peripheral edge of the growing tumor in proximity to the normal liver tissue may be effectively exposed to PLD and PEM by diffusion from the neighboring parenchyma, thus enabling a strong antitumor effect with metabolic shutdown of the tumor as seen by PET-CT.

Interestingly, there was a signal of differential gene expression profile between responders and nonresponders in the NanoString IO panel (Fig. 5). Because of the small sample size and the variable timing of tissue sampling, these results should be considered preliminary and requiring further correlative analysis in larger patient samples. The upregulation of IFN-stimulated genes in responder patients is interesting, as these genes increase the expression of major histocompatibility complex (MHC) molecules enabling antigen presentation to the immune system by MHC class I (44, 45). The expression of MHC class I antigens is variable in metastases and seems to be an important determinant of clinical response to cancer immunotherapy (46, 47). Exploring the expression of IFN-stimulated genes in tumor tissue samples before and after chemotherapy may provide a mechanistic insight in chemoimmunotherapy.

A proposed strategy of chemoimmunotherapy is that of priming the immune response with an initial course of ICD-inducers (particularly cisplatin or doxorubicin) followed in subsequent cycles by anti-PD1 treatment to enhance the sensitivity to PD1 blockade and avoid cytotoxic damage to activated T cells (48). Based on animal studies, we can predict that priming with PLD should work as well or better than with free doxorubicin (17). In fact, given the slow release of PLD and its mild myelosuppressive effect in clinical studies (20) and significant immunomodulatory effect in animal studies (24), concomitant treatment should work as well as priming and sequential treatment. Another strategy that we propose to potentiate the PLD–PEM combination, particularly for patients with rapidly PD approaching organ failure, is to add an ICD-inducer of rapid bioavailability, such as cyclophosphamide or low-dose cisplatin, to achieve a quick response and counterbalance the slow drug release kinetics of PLD. Yet, a different approach would involve patient selection based on biomarkers predicting response to immunotherapy. This last approach is supported by a recently published study in ER+ patients combining anti-PD1 with chemotherapy and demonstrating higher rates of pathologic responses to neoadjuvant treatment in PDL1-positive tumors with a high number of tumor-infiltrating lymphocytes (10). Another recent study in ER+, endocrine treatment– and CDK 4/6 inhibition–resistant MBC examined first-line chemotherapy with PEM and taxanes in 20 patients selected for a nonluminal gene expression profile using the PAM50 genomic assay and obtained an impressive clinical benefit rate (94%) and prolonged median OS (26 months) similar to that observed in this study (49). We would also argue for an approach based on interventions that can increase production of IFNγ and upregulate the IFN-stimulated genes and MHC class I molecules such as radiotherapy (50) or zoledronic acid, an aminobisphosphonate and activator of γδ T cells (51, 52).

In conclusion, the combination of PLD with PEM seems to be feasible and tolerable for extended treatment without unexpected toxicity. Compared with other chemoimmunotherapeutic combinatorial approaches, PLD and PEM have a relatively concordant PK which may facilitate synergistic activity. The encouraging activity of this regimen in patients with liver metastases should be further explored in phase II studies. When comparing with previous studies comprising ER+, endocrine–resistant patients with advanced breast cancer not receiving immunotherapy, the median survival of patients in this study seems to be notably longer. However, the small size of this study conducted in a single center along 6 years poses obvious limitations to the strength of the data which should be considered as hypothesis-generating until validation by a randomized study based on this nanoimmunotherapy approach.

## Supplementary Material

Supplement Table S-1 Supplement Table S-1: Serious Adverse Events (SAE)

Supplement Table S-2 Supplement Table S-2: Characteristics of Responders including prior and post-study therapies

Supplement Table S-3 Supplement Table S-3: Pharmacokinetic parameters of PEM in Patients (Pt) #2 and #3 after cycles 1 and 3

Supplement Table S-4 Supplement Table S-4: Biological Markers

Supplement Table S-5 Supplement Table S-5. Representativeness of Study Participants

Supplement Figure S-1 Study flow diagram

Supplement Figure S-2 Anti-tumor response

Supplement Figure S-3 Anti-tumor response

Supplement Figure S-4 Anti-tumor response

Supplement Figure S-5 Anti-tumor response

Supplement Figure S-6 Anti-tumor response

Supplement Figure S-7 Anti-tumor response

Supplement Figure S-8 Anti-tumor response

Supplement Figure S-9 Anti-tumor response

Supplement Figure S-10 Anti-tumor response

Supplement Figure S-11 Anti-tumor response

Supplement Figure S-12 Correlation of duration of response with survival

Supplement Figure S-13 Expression of immune checkpoint genes in responders (R) vs non-responders (NR)

## Data Availability

The datasets generated and/or analyzed during the current study are available from the corresponding author.
