# Supplementary material for: A Phase Ib Study of Chemoimmunotherapy with Pegylated Liposomal Doxorubicin and Pembrolizumab in Estrogen Receptor–Positive Metastatic Breast Cancer
Source: Cancer Res Commun. 2026 Jul 21;6(7):1738–49. doi: 10.1158/2767-9764.CRC-25-0539 (PMC13395262; doi:10.1158/2767-9764.CRC-25-0539)
Supplement: Supplement Table S-1 — Serious Adverse Events (SAE) [file crc-25-0539_supplement_table_s-1_suppst1.pdf]

Supplement Table S-1: Serious Adverse Events (SAE)

Pt #	Diagnosis (Grade)	Hospitalization	Resolved (Y/N)	Life-threatening (Y/N/Death)	Treatment-related NO/Possibly/Probably
1	Hepato-renal Failure (G. 5)	Yes	N	Death	NO
9	Hydronephrosis (G. 3)	Yes	N	Y	NO
17	Hemolytic Anemia (G. 4)	Yes	Y	Y	Probably (PEM)
18	Hepatitis (G. 4)	Yes	Y	N	Possibly (PEM)
18	Arm wound infection/Nocardia abscess (G. 3)	Yes	Y	Y	NO
22	Hepatitis (G. 4)	Yes	Y	N	Possibly (PEM)
25	Cholecystitis M/P (G. 3)	Yes	Y	Y	NO
34	UTI/Urosepsis (G. 3)	Yes	Y	N	NO
35	Facial Palsy (G. 3)	Yes	N	N	NO
36	Ascites/Dehydration (G. 4)	Yes	Y	Y	NO
