# Supplementary material for: A Phase Ib Study of Chemoimmunotherapy with Pegylated Liposomal Doxorubicin and Pembrolizumab in Estrogen Receptor–Positive Metastatic Breast Cancer
Source: Cancer Res Commun. 2026 Jul 21;6(7):1738–49. doi: 10.1158/2767-9764.CRC-25-0539 (PMC13395262; doi:10.1158/2767-9764.CRC-25-0539)
Supplement: Supplement Table S-2 — Characteristics of Responders including prior and post-study therapies [file crc-25-0539_supplement_table_s-2_suppst2.pdf]

**Supplement Table S-2:** Characteristics of Responders including prior and post-study therapies

| Patient # | Aromatase Inhibitor | SERD | CDK 4,6 inhibitor | Other | Chemo Lines (n)[a] | Prior Dox[b] | Response Duration (m) | % decrease of long diameter in target lesion | Post-study treatment[c] | Survival (m)[d] |
|---|---|---|---|---|---|---|---|---|---|---|
| 5 (Fig. S-2) | Y | Y | Y | N | 2 | N | 11 | 40% (axilla) | Gem, CMF, PTX, Carbo, VNR | 39.0 |
| 10 (Fig. S-3) | Y | N | Y | Tamoxifen | 2 | Y | 15 | 76% (liver) | PTX, Carbo, Gem | 44.7 |
| 15 (Fig. S-4) | Y | Y | Y | N | 2 | Y | 5 | 51% (liver) | DTX, Cap, CMFVP | 26.4 |
| 17 (Fig. S-5) | Y | Y | Y | Tamoxifen/ Everolimus | 3 | N | 20 | 24% (retro-crural) | VNR, PTX, Dox, Gem, Carbo, Tam, Ribociclib | 50.6**+** |
| 18 (Fig. S-6) | Y | N | Y | Tamoxifen | 4 | Y | 15 | 100% (liver) | Carbo, CMFVP | 27.6 |
| 21 (Fig. S-7) | Y | N | Y | N | 2 | N | 8 | 76% (liver) | Carbo, Gem | 13.4 |
| 23 (Fig. S-8) | Y | Y | Y | Everolimus | 3 | N | 6 | 68% (liver) | Vinorelbine | 12.0 |
| 30 (Fig. S-9) | Y | Y | Y | Alpelisib | 2 | N | 12.5 | 90% (liver) | Dox, CMF | 29.2**+** |
| 32 (Fig.S-10) | Y | Y | Y | Tamoxifen | 3 | Y | 9 | 71% (mediastinum) | PTX, Gem, Carbo | 27.0**+** |
| 37 (Fig. S-11) | Y | N | Y | N | 2 | Y | 4.6+ | 32% (thyroid) | None | 9.6**+** |

a. Including Neo/Adjuvant chemotherapy.
b. Adjuvant or neoadjuvant
c. Abbreviation of agents: Cap=Capecitabine; Carbo=Carboplatin; CMF=cyclophosphamide, methotrexate, 5-fluouracyl; CMFVP= cyclophosphamide, methotrexate, 5-fluouracyl, vincristine, prednisone; Dox=doxorubicin; DTX=docetaxel; Gem=gemcitabine; PTX=paclitaxel; Tam=tamoxifen; VNR=vinorelbine.
d. Patients alive at data lock are marked with **+**
