# Supplementary material for: A Phase Ib Study of Chemoimmunotherapy with Pegylated Liposomal Doxorubicin and Pembrolizumab in Estrogen Receptor–Positive Metastatic Breast Cancer
Source: Cancer Res Commun. 2026 Jul 21;6(7):1738–49. doi: 10.1158/2767-9764.CRC-25-0539 (PMC13395262; doi:10.1158/2767-9764.CRC-25-0539)
Supplement: Supplement Table S-3 — Pharmacokinetic parameters of PEM in Patients (Pt) #2 and #3 after cycles 1 and 3 [file crc-25-0539_supplement_table_s-3_suppst3.pdf]

Supplement Table S-3: Pharmacokinetic parameters of PEM in Patients (Pt) #2 and #3 after cycles 1 and 3

	Cmax mg/L	$AUC_{o\text{-}last}$ mg*day/L	T½ days	CL mL/day	Vcc L	C_{trough}[1] mg/L
Pt. #2 Cycle 1	67.15	887	19.25	225	3.0	38.9
Pt. #2 Cycle 3	148.0	2132	18.8	94	1.35	87.4
Pt. #3 Cycle 1	99.3	1005	9.4	199	2.0	28.0
Pt. #3 Cycle 3	227.0	2899	14.9	69	0.9	89.2

1. C_{trough} measured on cycle day 21-22 immediately before next cycle
