# Supplementary material for: A Phase Ib Study of Chemoimmunotherapy with Pegylated Liposomal Doxorubicin and Pembrolizumab in Estrogen Receptor–Positive Metastatic Breast Cancer
Source: Cancer Res Commun. 2026 Jul 21;6(7):1738–49. doi: 10.1158/2767-9764.CRC-25-0539 (PMC13395262; doi:10.1158/2767-9764.CRC-25-0539)
Supplement: Supplement Table S-4 — Biological Markers [file crc-25-0539_supplement_table_s-4_suppst4.pdf]

Supplement Table S-4: Biological Markers (when cell empty, test not done)

Patient #	MMR / MSI	PDL1	TMB	Response
1	Proficient	Negative		NE (fast PD)
2	Proficient	Negative		PD
3				SD
4	Proficient	Negative		SD
6	Proficient	Negative		SD
9	Proficient	Negative		PD
10	Proficient	Positive (CPS=17)		PR/CR
11	Proficient		Low (3 mut/Mb)	NE (fast PD)
12	Proficient	Negative		PD
13	Proficient			PD
15	Proficient	Negative		PR
16	Proficient		Low (3 mut/Mb)	NE (fast PD)
17	Proficient			PR
28	Proficient		Low (7 mut/Mb)	SD
30	Proficient			PR
33		Negative		PD
