# Supplementary material for: A Phase Ib Study of Chemoimmunotherapy with Pegylated Liposomal Doxorubicin and Pembrolizumab in Estrogen Receptor–Positive Metastatic Breast Cancer
Source: Cancer Res Commun. 2026 Jul 21;6(7):1738–49. doi: 10.1158/2767-9764.CRC-25-0539 (PMC13395262; doi:10.1158/2767-9764.CRC-25-0539)
Supplement: Supplement Table S-5 — Representativeness of Study Participants [file crc-25-0539_supplement_table_s-5_suppst5.pdf]

Supplement Table S-5. Representativeness of Study Participants

Cancer type(s)/subtype(s)/stage(s)/condition	Estrogen Receptor Positive Breast Cancer (ER+)
Considerations related to:	
Sex	ER+ breast cancer, as all other subtypes of breast cancer, is a predominantly female disease and is rare in men. All 35 patients in this study are women.
Age	The median age of our study patients at the time of accrual to study (Stage 4 disease) is 60 years with a broad range (36-91).
Race/ethnicity	In Israel, Blacks represent <2% of the population and East Asian are even a much smaller fraction. All our 35 patients are Caucasian, either European or MENA. 29 patients (83%) identified as Jews or non-specified. Six of them are Arabs (17%),
Geography	Given that Israel is a very small country, this factor is not relevant.
Overall representativeness of this study	The age and ethnic distribution of our study reflect well the Israeli population. However, at the global level, it certainly falls below the required representativeness. Since this is a small phase 1b study, the relevance of the representativeness factor will surely be addressed in further larger studies examining the combination therapy studied here.
