# Supplementary figures and images for: A Phase Ib Study of Chemoimmunotherapy with Pegylated Liposomal Doxorubicin and Pembrolizumab in Estrogen Receptor–Positive Metastatic Breast Cancer
Source: Cancer Res Commun. 2026 Jul 21;6(7):1738–49. doi: 10.1158/2767-9764.CRC-25-0539 (PMC13395262; doi:10.1158/2767-9764.CRC-25-0539)

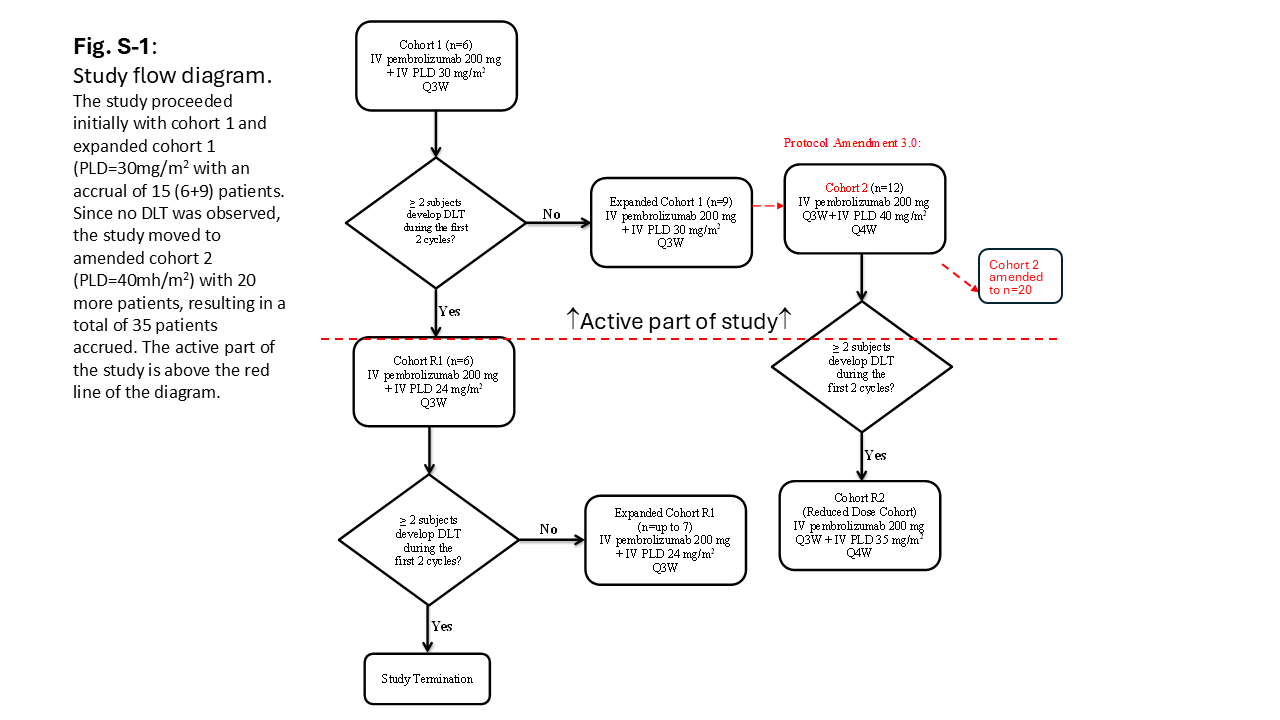

Supplement: Supplement Figure S-1 — Study flow diagram [file crc-25-0539_supplement_figure_s-1_suppsf1.png]

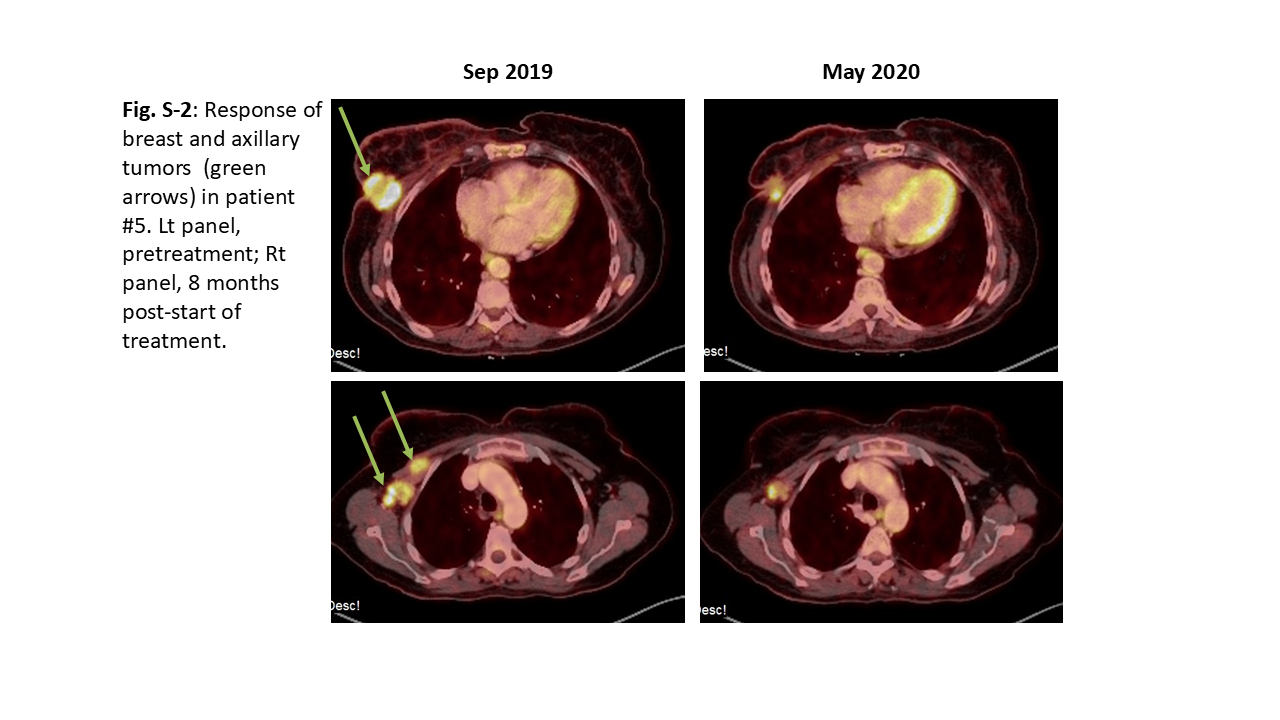

Supplement: Supplement Figure S-2 — Anti-tumor response [file crc-25-0539_supplement_figure_s-2_suppsf2.png]

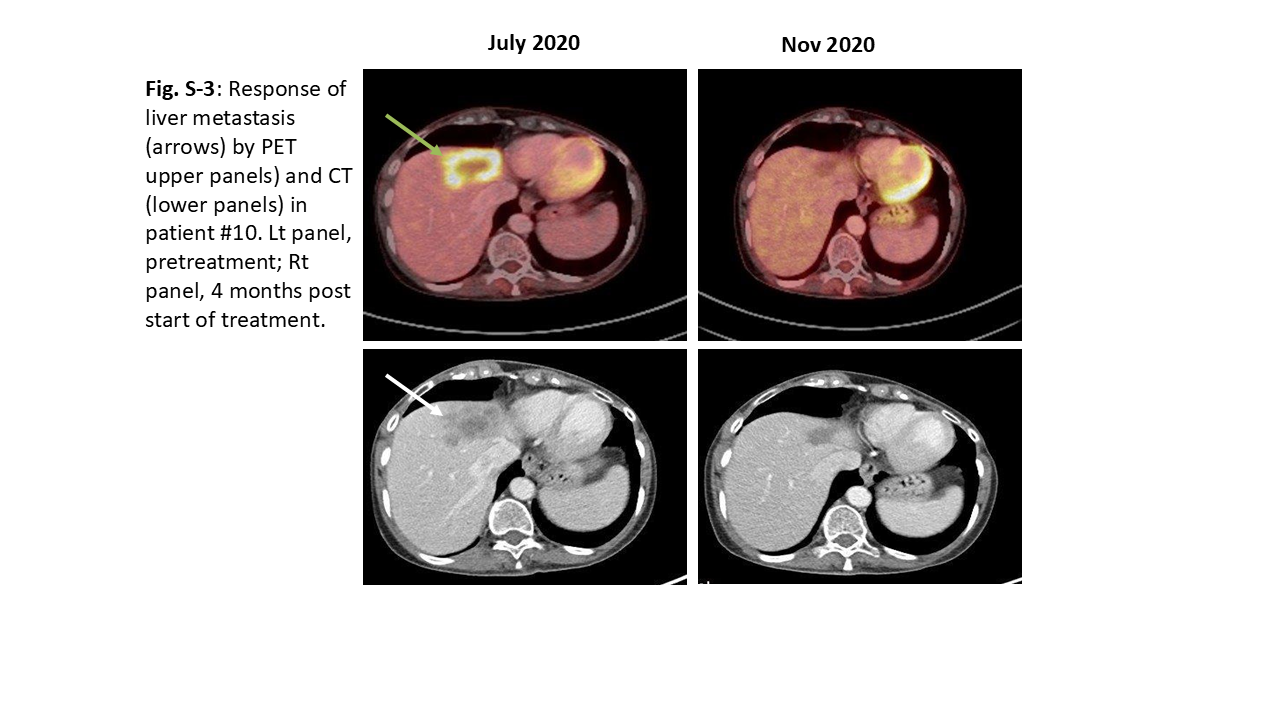

Supplement: Supplement Figure S-3 — Anti-tumor response [file crc-25-0539_supplement_figure_s-3_suppsf3.png]

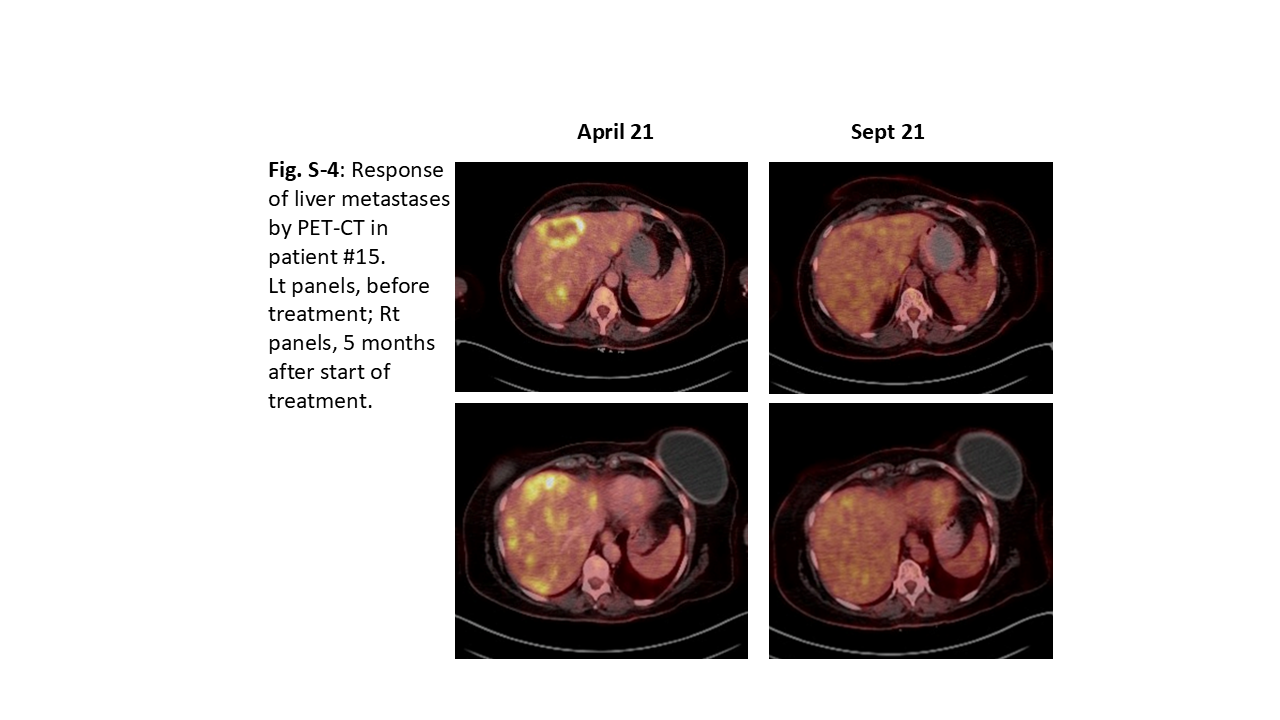

Supplement: Supplement Figure S-4 — Anti-tumor response [file crc-25-0539_supplement_figure_s-4_suppsf4.png]

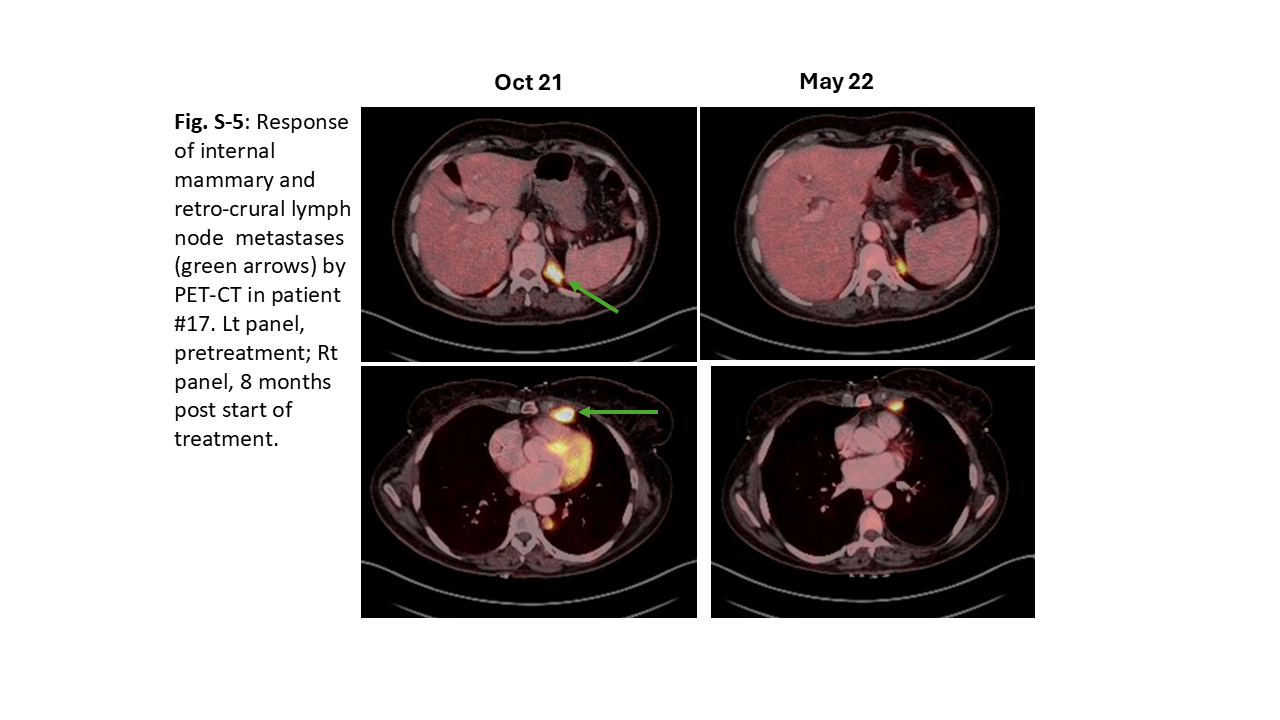

Supplement: Supplement Figure S-5 — Anti-tumor response [file crc-25-0539_supplement_figure_s-5_suppsf5.png]

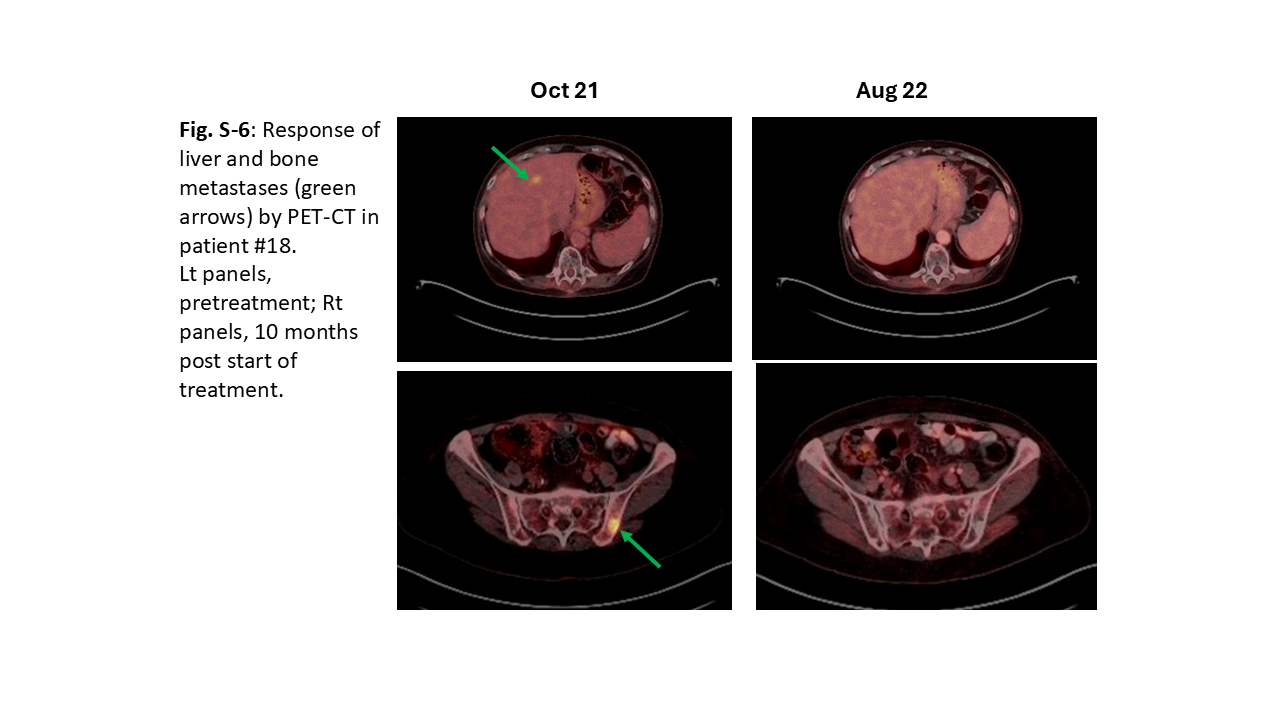

Supplement: Supplement Figure S-6 — Anti-tumor response [file crc-25-0539_supplement_figure_s-6_suppsf6.png]

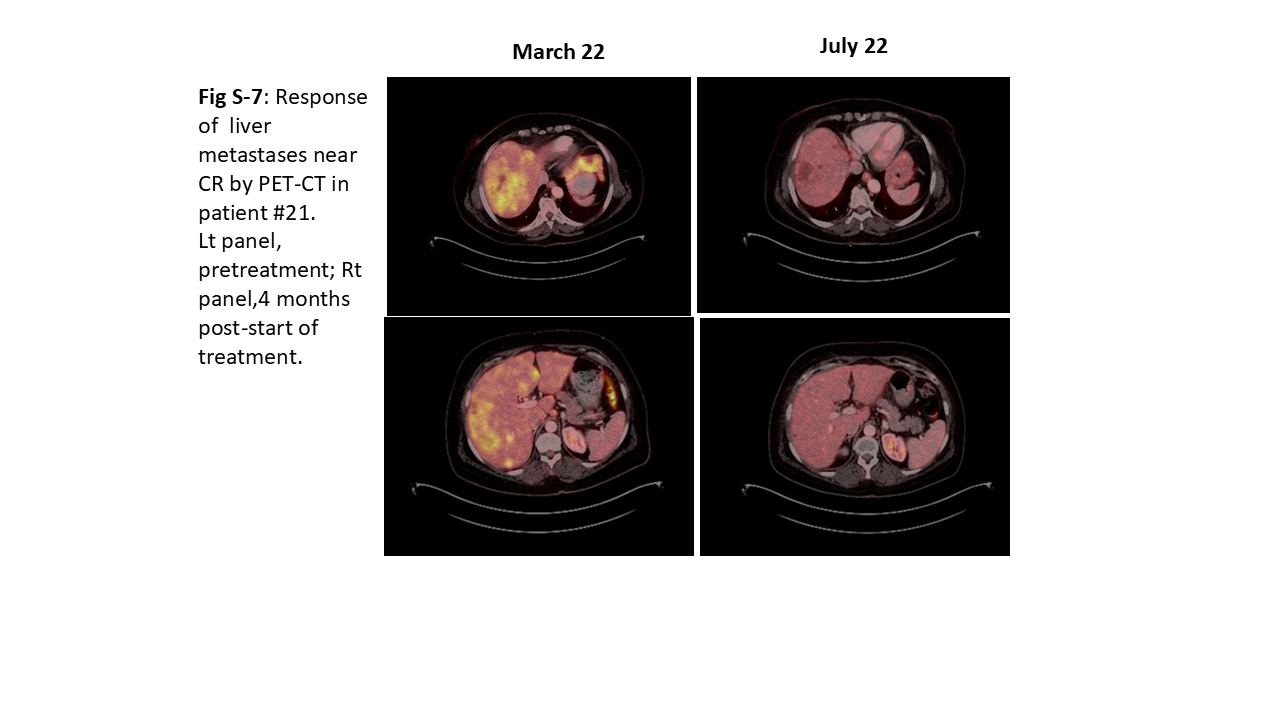

Supplement: Supplement Figure S-7 — Anti-tumor response [file crc-25-0539_supplement_figure_s-7_suppsf7.png]

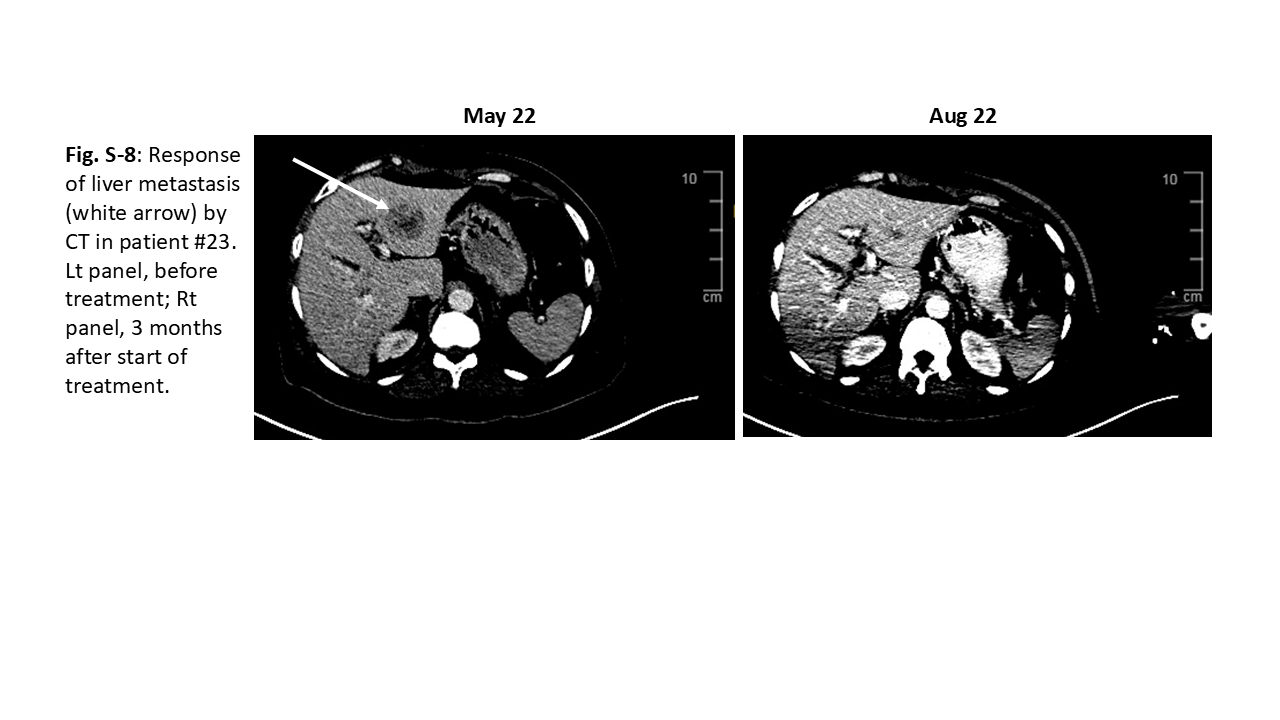

Supplement: Supplement Figure S-8 — Anti-tumor response [file crc-25-0539_supplement_figure_s-8_suppsf8.png]

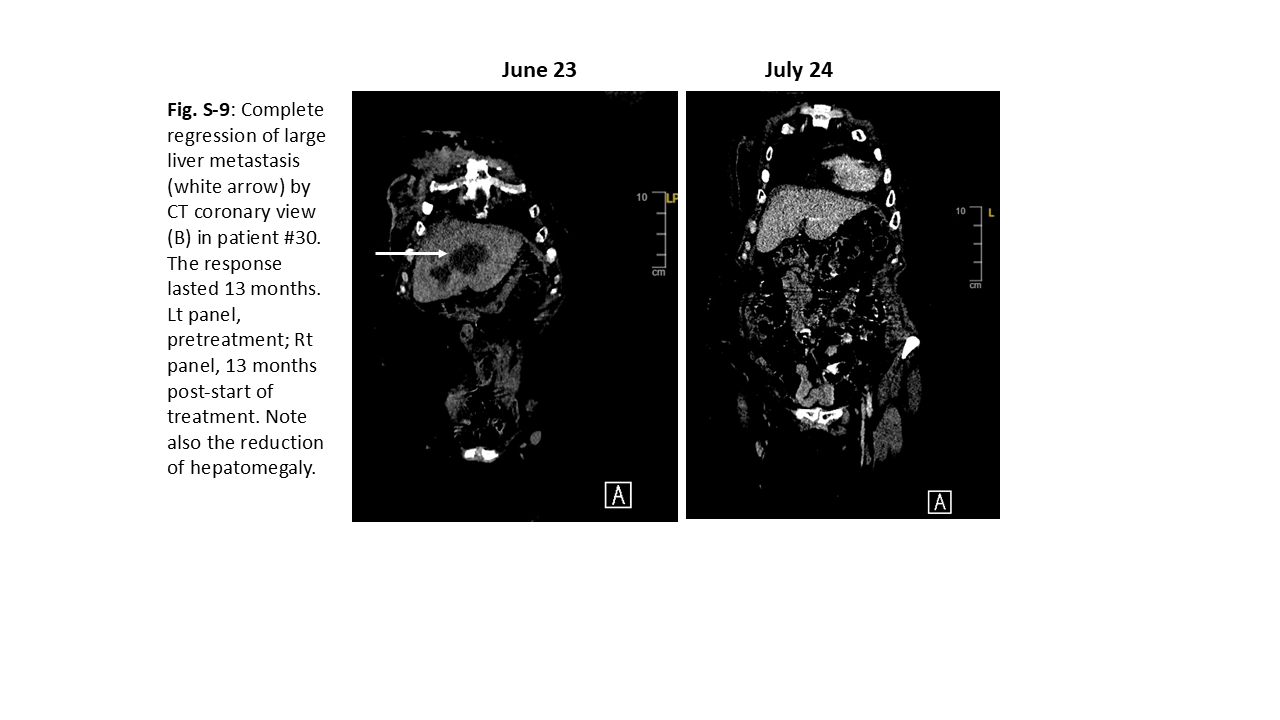

Supplement: Supplement Figure S-9 — Anti-tumor response [file crc-25-0539_supplement_figure_s-9_suppsf9.png]

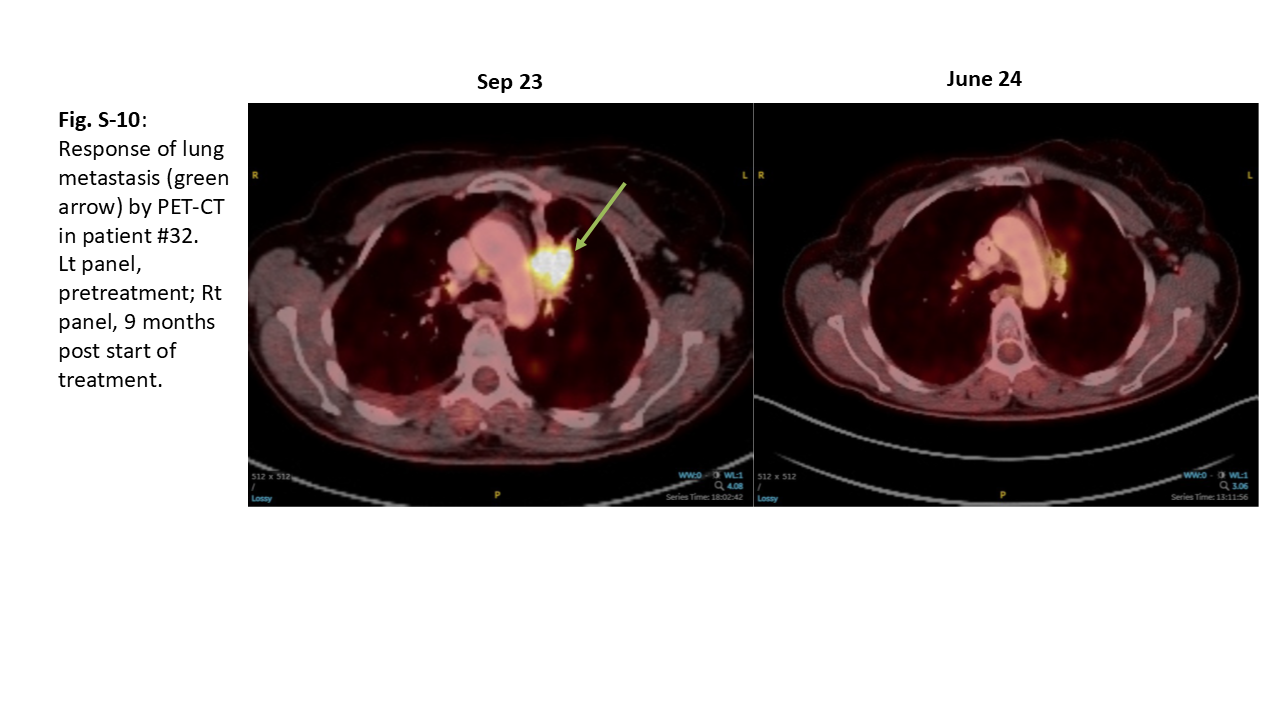

Supplement: Supplement Figure S-10 — Anti-tumor response [file crc-25-0539_supplement_figure_s-10_suppsf10.png]

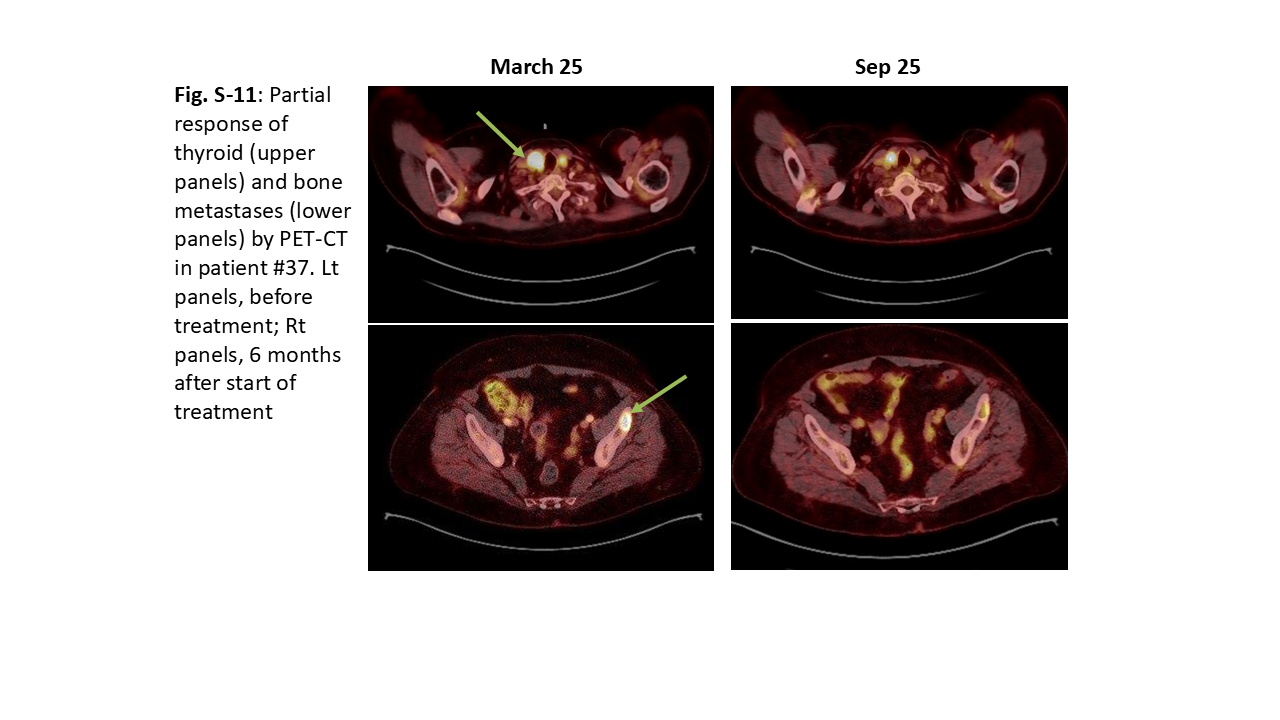

Supplement: Supplement Figure S-11 — Anti-tumor response [file crc-25-0539_supplement_figure_s-11_suppsf11.png]

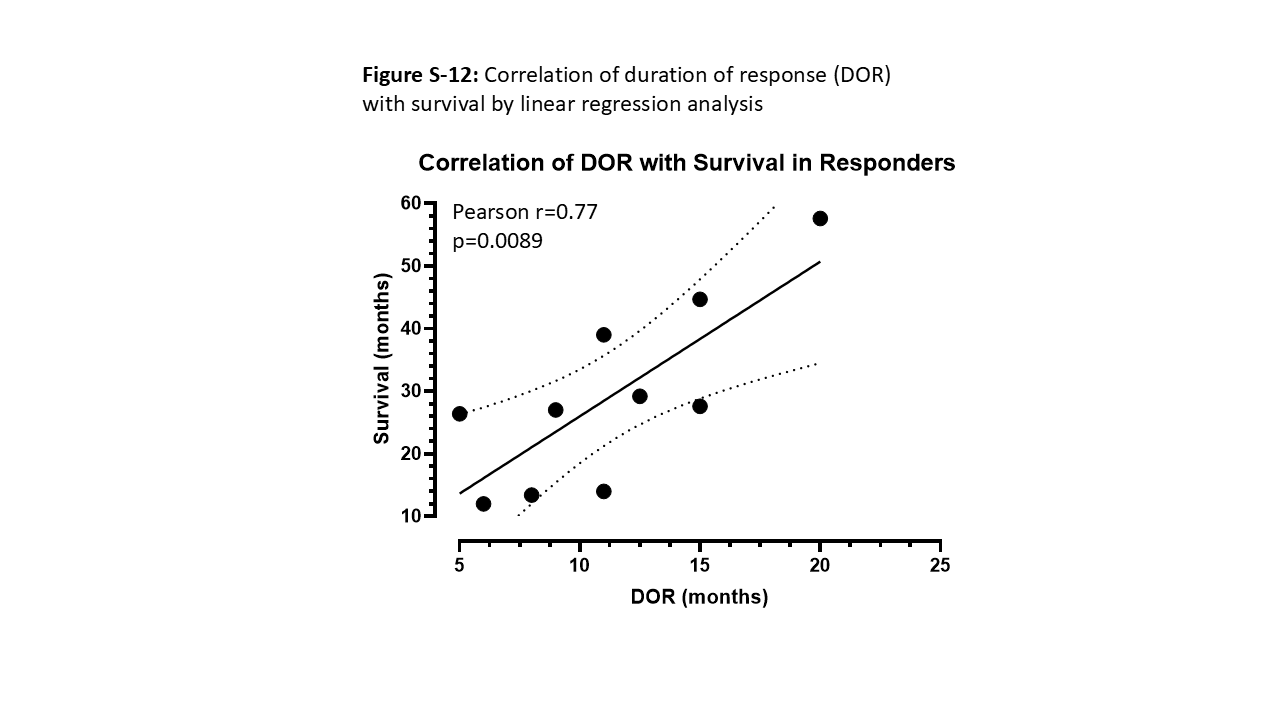

Supplement: Supplement Figure S-12 — Correlation of duration of response with survival [file crc-25-0539_supplement_figure_s-12_suppsf12.png]

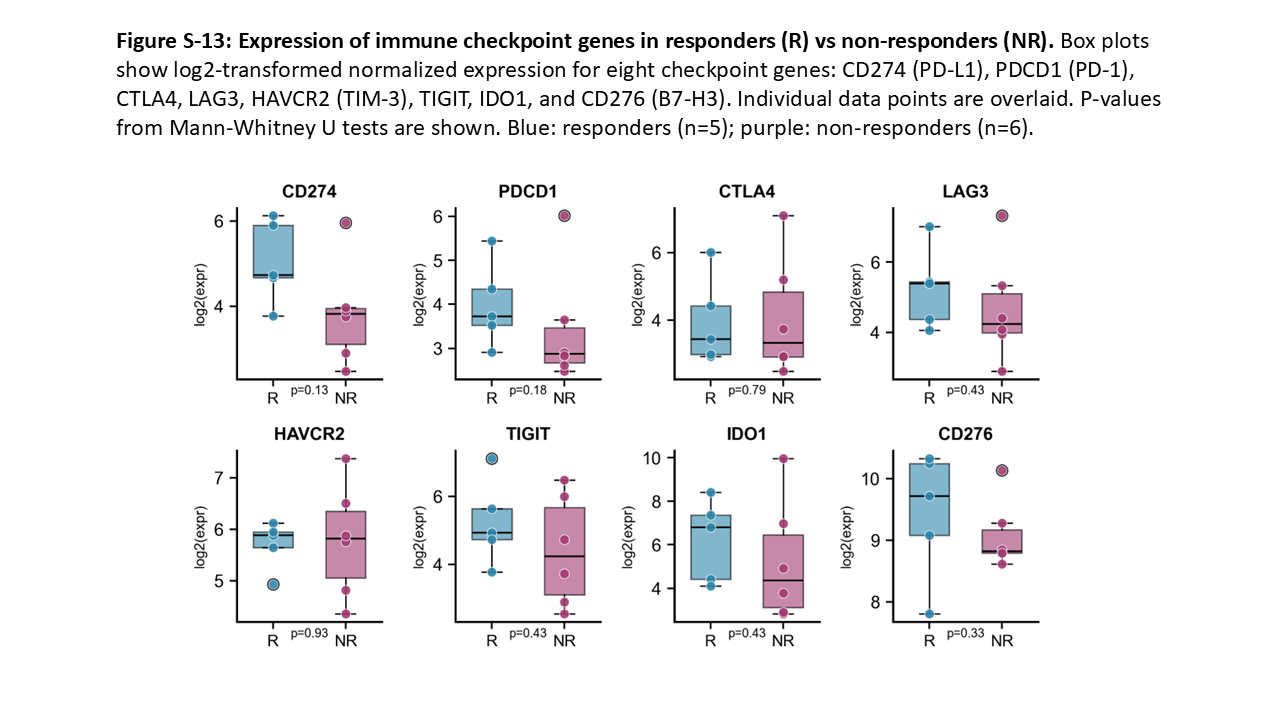

Supplement: Supplement Figure S-13 — Expression of immune checkpoint genes in responders (R) vs non-responders (NR) [file crc-25-0539_supplement_figure_s-13_suppsf13.png]
